# Implicit Motivational Impact of Pictorial Health Warning on Cigarette Packs

**DOI:** 10.1371/journal.pone.0072117

**Published:** 2013-08-15

**Authors:** Eliane Volchan, Isabel A. David, Gisella Tavares, Billy M. Nascimento, Jose M. Oliveira, Sonia Gleiser, Andre Szklo, Cristina Perez, Tania Cavalcante, Mirtes G. Pereira, Leticia Oliveira

**Affiliations:** 1 Institute of Biophysics Carlos Chagas Filho, Universidade Federal do Rio de Janeiro, Rio de Janeiro, Brazil; 2 Biomedical Institute, Universidade Federal Fluminense, Niteroi, Brazil; 3 Institute of Psychiatry, Universidade Federal do Rio de Janeiro, Rio de Janeiro, Brazil; 4 Coordination for Prevention and Surveillance, Brazilian National Cancer Institute, Rio de Janeiro, Brazil; 5 Executive Secretariat of the National Commission for FCTC Implementation in Brazil, Brazilian National Cancer Institute, Rio de Janeiro, Brazil; University of California, United States of America

## Abstract

**Objective:**

The use of pictorial warning labels on cigarette packages is one of the provisions included in the first ever global health treaty by the World Health Organization against the tobacco epidemic. There is substantial evidence demonstrating the effectiveness of graphic health warning labels on intention to quit, thoughts about health risks and engaging in cessation behaviors. However, studies that address the implicit emotional drives evoked by such warnings are still underexplored. Here, we provide experimental data for the use of pictorial health warnings as a reliable strategy for tobacco control.

**Methods:**

Experiment 1 pre-tested nineteen prototypes of pictorial warnings to screen for their emotional impact. Participants (n = 338) were young adults balanced in gender, smoking status and education. Experiment 2 (n = 63) tested pictorial warnings (ten) that were stamped on packs. We employed an innovative set-up to investigate the impact of the warnings on the ordinary attitude of packs’ manipulation, and quantified judgments of warnings’ emotional strength and efficacy against smoking.

**Findings:**

Experiment 1 revealed that women judged the warning prototypes as more aversive than men, and smokers judged them more aversive than non-smokers. Participants with lower education judged the prototypes more aversive than participants with higher education. Experiment 2 showed that stamped warnings antagonized the appeal of the brands by imposing a cost to manipulate the cigarette packs, especially for smokers. Additionally, participants’ judgments revealed that the more aversive a warning, the more it is perceived as effective against smoking.

**Conclusions:**

Health warning labels are one of the key components of the integrated approach to control the global tobacco epidemic. The evidence presented in this study adds to the understanding of how implicit responses to pictorial warnings may contribute to behavioral change.

## Introduction

The four leading non-communicable diseases, cardiovascular disease, cancer, chronic respiratory disease and diabetes, represent a leading threat to human health and development. According to the World Health Organization (WHO) [1], these four diseases are the world’s biggest killers, causing 60% of all deaths globally and 80% in low- and middle-income countries. Up to 80% of heart disease, stroke, type 2 diabetes and over a third of cancers could be prevented by eliminating shared risk factors, mainly tobacco use, unhealthy diet, physical inactivity and the harmful use of alcohol. In fact, in 2011, tobacco control was identified as the “most urgent and immediate priority” to reduce non-communicable diseases [2].

The Framework Convention on Tobacco Control, the first international public health treaty initiated by the World Health Organization, represents the most significant tobacco control initiative to date. The treaty considers tobacco an epidemic, and the transnational tobacco corporations, by their actions to maximize global tobacco consumption, as a “vector” of this epidemic. The use of prominent pictorial health warning labels on cigarette packages is one of the provisions included in the Framework Convention on Tobacco Control (FCTC) [3]. Adoption of the FCTC recommendations has been partially prevented by political and judicial obstacles orchestrated by tobacco corporations [4], including denying their advantage and effectiveness [5]. For example, a supposed lack of evidence that graphic health warning labels would act on behalf of tobacco control was used as allegation for the unconstitutionality of this FCTC recommendation in the United States [6].

There is substantial evidence favoring the effectiveness of graphic health warnings on intentions to quit, thoughts about health risks and engaging in cessation behavior [7]–[10]. However, an approach that addresses the implicit emotional drives evoked by these warnings is underexplored. Indeed, a recent review on health warning labels noted the need to fill a remaining research gap, the one on implicit responses evoked by exposure to pictorial warnings [11]. A vast body of research on the neurobiology of emotion demonstrates that looking at pictures implicitly affects attitudes and behaviors; pleasant and unpleasant pictures evoke emotions of attraction and aversion, respectively, the magnitudes of which correlate with the strength of the affective content [12]. The tobacco industry has long employed pleasant images to attract consumers. Grounded on studies of the emotional impact of picture viewing, graphic pictorial warnings are a clear way to dismantle the pleasurable appeal and attenuate the implicit lure of cigarette packs. As an additional benefit, the graphic warnings aim to facilitate the cognitive appraisal of the depicted information about smoking hazards.

The major aim of the present study is to extend the existing literature by providing experimental data on implicit drives. Furthermore, we present evidence that pictorial health warnings are a reliable strategy for policies regarding the control of tobacco products.

The study took place in Brazil, the second country (after Canada) to introduce pictorial warnings on cigarette packs. Cigarette packages and other tobacco-related products in Brazil must carry health warnings containing color pictures covering 100% of one face of the package. The smoking rate in Brazil has decreased by approximately 50% from 1989 to 2008 [13]. Brazil implemented the first set of pictorial warnings in 2002 and the second set in 2004.

For the third set, released in 2009, the whole process (from planning and producing to testing) was grounded on a pioneering strategy that involved the union of expertise on experimental psychology, neuroscience, public health and design [14]. Here, we present experimental testing of warnings in the prototype phase (Experiment 1) and when released on cigarette packs (Experiment 2).

## Experiment 1

The casings of cigarettes are carefully elaborated to attract consumers and to mask the harmful effects of tobacco. Pictorial warning labels stamped on packages are sought to undo this appeal and uncover the health risks associated with the product.

Lang and collaborators developed a standard catalog of pictures, the International Affective Picture System [15], to use in psychophysiological studies of emotion; and a scale, the Self-Assessment Manikin [16], to directly assess the pleasantness/unpleasantness (hedonic valence) and emotional arousal associated with each picture. The ratings obtained from this scale correlate with several physiological and behavioral reactions to the pictures and can be considered, to some extent, an index of the implicit emotional activation of the appetitive (“attraction”) and defensive (“rejection”) systems [12].

Among the unpleasant and arousing pictures, it is well established that those depicting injuries to the body are the most impactful; they significantly capture attention, interfere with other tasks and induce defensive-like reactions (see [17]–[21]). These findings motivated the selection of graphic scenes of tobacco-related harms to produce nineteen pictorial prototypes for the third set of Brazilian warnings. The experimental evaluation of these prototypes was based on the valence/arousal protocol for rating affective pictures [15].

### Methods

#### Ethics statement

This study was approved by the Ethics Review Board of the Federal University of Rio de Janeiro (Brazil). All participants provided written informed consent before assessment.

#### Participants

Volunteers (n = 338) from 18 to 24 years old were recruited from a registry of movie extras. Selection criteria aimed to balance the sample in gender (168 women), smoking/non-smoking status (154 smokers), and education (incomplete elementary school, n = 99; high school, n = 117; university, n = 122).

The connection between the experimental session and the tobacco control program was not made explicit to the participants.

#### Stimuli and apparatus

Prototype warnings consisted of 19 digital pictures. In addition to the prototypes tested, we employed 75 pleasant, neutral and unpleasant pictures taken from the “International Affective Picture System” [15] catalog.

The experiment was conducted in a dimly lit room with comfortable desks placed in rows in front of a slide projection screen. Desks were arranged in such a manner that the screen was perfectly visible to every participant. A computer-projector system controlled the presentation timing of each picture. The presentation order of the 94 pictures was pseudo-randomized. No more than 40 participants performed the test simultaneously.

#### Evaluation scale

Participants were asked to rate each picture along the dimensions of hedonic valence and emotional arousal using the paper-and-pencil version of the Self- Assessment Manikin [16]. The scale of the hedonic valence dimension is composed of pictorial drawings of manikins with expressions ranging from “smiling-happy” to “frowning-unhappy”. For the arousal dimension, the expressions of the manikins range from an excited, wide-eyed figure to a relaxed, sleepy figure. Before the start of the experimental session, a didactic video explained the upcoming task.

#### Procedure

Each rating trial began with a preparation slide (“Get ready to rate the next image”) that lasted for 3 s and was followed by a 6 s picture observation period. During the next 10 s, participants were asked to rate the picture along the dimensions of hedonic valence and emotional arousal using the paper-and-pencil version of the Self- Assessment Manikin scales [16].

#### Data analysis

For analysis purposes, the ratings in the hedonic valence dimension were converted to numbers ranging from −4 (extremely unpleasant) to +4 (extremely pleasant), with 0 being neutral. The ratings in the emotional arousal dimension were converted to numbers ranging from 1 (low arousal) to 9 (high arousal). The average ratings from the participants were computed for each prototype and displayed in Cartesian coordinates with valence along the y-axis and arousal along the x-axis (see [22]). The x and y coordinates for each prototype were mathematically combined and transformed into a vector representing the degree of aversion attributed to each picture. The magnitudes of the vectors for each prototype were compared between SMOKERS and NON-SMOKERS and between MEN and WOMEN using Student’s *t*-test. To compare vector magnitudes for each prototype based on the participants’ educational levels (ELEMENTARY/HIGH SCHOOL/UNIVERSITY), we used a repeated measures ANOVA with a Fischer post-hoc test.

In all analyses, the statistical threshold for significance was a p-value of 0.05.

### Results

All prototypes were rated as unpleasant. The mean valence for the prototypes was −2.3 (±0.58) (range: −1.4 to −3.5) and mean arousal was 5.5 (±0.50) (range: 4.7 to 6.5).

WOMEN judged the warning prototypes more aversive than MEN (Student’s *t*-test, t (18) = 4.02; p<0.05). SMOKERS judged them more aversive than NON-SMOKERS (Student’s *t*-test, t (18) = 3.39; p<0.05). Education (ELEMENTARY/HIGH SCHOOL/UNIVERSITY) also affected the ratings (F (2, 36) = 15.06; p<0.05). Post-hoc analyses showed that the three educational levels differed significantly from each other. Participants with incomplete high school or incomplete elementary school educations rated the prototypes as more aversive than the participants with the highest educational level.

Judgments of emotional impact were a major criterion for the selection of the pictures to be used as the third set of warnings on cigarette packages in Brazil. These warning pictures were tested in Experiment 2.

## Experiment 2

Researchers have posited that all experience is continually evaluated as either positive or negative. Any positively evaluated stimulus produces motor preparedness for bringing it closer, while negatively evaluated stimuli produce motor preparedness to repel it [23]–[25].

The relevance of cigarette packages as a primary instrument for tobacco promotion has increased, and packages have become more attractive. Smokers usually manipulate the pack every time they smoke (20 cigarettes a day corresponds to 7300 manipulations during a year). Could graphic warnings interfere with the automatic motor preparation to manipulate attractive packs?

We addressed this question by testing the third set of Brazilian warnings on the packs. We applied an innovative experimental set-up [26] which allowed assessment of reactions when individuals interact with emotion-laden objects, such as a cigarette pack. Through this approach, we investigated the interference of these warnings on the ordinary attitude of cigarette pack’s manipulation, expected to be more relevant to smokers (as compared to non-smokers). Additionally, we gathered each participant’s judgment of the warning’s emotional strength and the ability of the warning to prevent smoking and promote smoking cessation.

### Methods

#### Ethics statement

This study was approved by the Ethics Review Board of the Federal University of Rio de Janeiro (Brazil). All participants provided written informed consent before assessment.

#### Participants

Participants were recruited through posters distributed on the University campus. Tobacco users among young adults in Brazil are less than 15% [27]. To increase the percentage of smokers in the sample, an additional interview was performed prior to scheduling the experimental session. The final sample had 63 participants with a mean age of 22.5 (±5.23) years old, of which 55% were women and 29% were current smokers.

#### Stimuli

Stimuli comprised 20 cigarette packs. Only one face of the pack, displaying either a warning image (n = 10) or a brand image (n = 10), was visible to the participants during each test (see Apparatus for more details). The warning images used were from the 2009 Brazilian set [14]. For the experiment, the images were stamped on the packs and were devoid of any text. Cigarette brands were selected so that they matched the warnings in color content.

#### Evaluation scales

Valence and arousal ratings for each warning displayed on the packs were assessed by the Self-Assessment Manikin [16].

Based on Fong et al. [28], the effectiveness of the warning for smoking prevention and for smoking cessation were separately evaluated on 10-point scales ranging from “not effective at all” to “very effective”.

#### Apparatus

**Figure 1:**
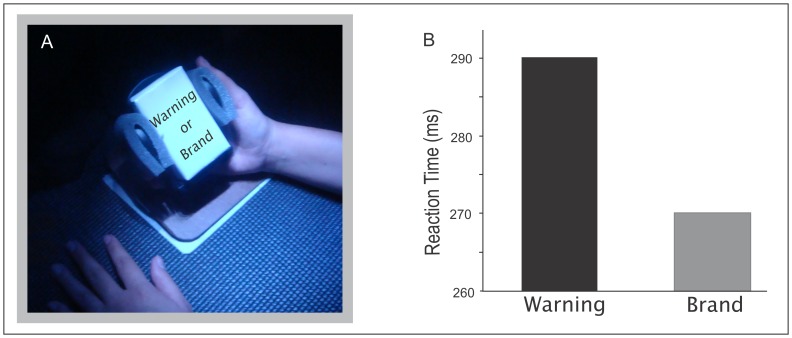
Interference of warnings on the manipulation of cigarette packs. (A) The participant grabs a holder with his/her dominant hand. Unseen by the participant, the experimenter places a cigarette pack on the holder, exposing either the brand face or the warning face. Upon illumination, the participant quickly flexes the arm. The latency between illumination and action (reaction time) is measured. (B) Reaction times for smokers. Reaction time when the warning face is exposed (left column of the graph) is significantly longer compared to when the brand face is exposed (right column). The results reveal that warnings impose a significant cost to the manipulation of the pack.

The experiment was conducted in a dimly lit room and employed the “Box for Interaction with Objects” (BIO) [26], a device designed to trigger an almost instantaneous onset of an object’s (cigarette pack) appearance. The BIO is a prism-shaped hollow box. The participant was seated facing the front of the BIO with the arms resting on its inner base. A reflective film prevented the participant from seeing the pack inside the BIO unless it is illuminated from within. The experimenter sat behind the BIO and controlled the visibility by turning the internal lights on and off. Cigarette packs were placed on a holder so that only the brand face or the warning face was exposed during each test (Figure 1A).

#### Procedure

Each test began with the participant resting the dominant hand on a weight sensor, keeping the holder in his/her hand. With the internal lights of the BIO turned off, the experimenter inserted a cigarette pack into the holder. Upon its illumination (see Figure 1A), the participant was instructed to quickly flex the arm, bringing the pack closer to his/her body. The release of the weight sensor signaled the start of the movement, allowing measurement of the reaction time. When the participant returned the holder to the initial position, the experimenter turned off the internal lights and changed the cigarette pack, and a new test began using a different pack. The sequence of appearance of the 20 different packs (displaying warnings or brands) was randomized prior to each experimental session.

After the behavioral session, there was a short interval to change the setup for the evaluation session. A frame designed to accommodate one pack was fixed inside the BIO. With the internal lights off, the experimenter placed a pack displaying a warning into the frame. A booklet positioned next to the frame was used to record the participant’s ratings. The warning face was made visible for approximately 6 seconds, subsequently the participant filled out the evaluation. The experimenter then turned off the internal lights, changed the cigarette pack and a new test began.

#### Data analysis

For the behavioral session, the parameter examined was the latency (reaction time) to start the movement of bringing the pack closer to the body. For each participant, we determined the median reaction time for warnings and for brands. Separate tests were conducted for smokers and non-smokers. As the data did not show a normal distribution, we used the Wilcoxon matched-pairs signed-ranks test for the analysis.

For the evaluation session, valence (ranging from −4 to +4) and arousal (ranging from 1 to 9) ratings attributed to each warning were averaged across participants and displayed in Cartesian coordinates with the mean valence on the y-axis and mean arousal on the x-axis. These values were combined and transformed into a vector whose magnitude indicated the degree of aversion attributed to each warning. For each warning, we also computed the average ratings attributed to its effectiveness in smoking prevention and its effectiveness in smoking cessation.

The degree of aversion (combination of arousal and valence ratings) of each warning (AVERSION) was compared with judgments of its effectiveness in smoking prevention (PREVENTION), and in smoking cessation (CESSATION); using Pearson’s correlations. Separate tests were conducted for smokers and non-smokers.

The threshold of statistical significance employed for all analyses was a p-value of 0.05.

### Results

Behavioral tests revealed that smokers were significantly slower to bring packs close to the body when showing warnings compared to when showing brands (Z = 2.11; p<0.05) (Figure 1B). For smokers, who habitually manipulate cigarette packs, stamped warnings antagonized the appeal of the brands. No significant difference was found for non-smokers (Z = 0.23; p = 0.82).

Evaluative tests revealed that judgments of the degree of aversion (AVERSION) correlated with judgments of efficacy against smoking (CESSATION/PREVENTION). For smokers, AVERSION correlated with CESSATION (r = 0.81, p<0.05) and with PREVENTION (r = 0.86, p<0.05). For non-smokers, AVERSION correlated with CESSATION (r = 0.67, p<0.05) and showed a trend toward correlation with PREVENTION (r = 0.57, p = 0.09). These results indicate that the more aversive a warning, the more it is perceived as effective against smoking.

## General Discussion

The World Health Organization Framework Convention on Tobacco Control [3] was developed in response to the globalization of the tobacco epidemic. The treaty was guided by the principle that “Every person should be informed of the health consequences, addictive nature and mortal threat posed by tobacco consumption and exposure to tobacco smoke…” [3]. In Article 11 [3], the treaty included effective health warnings as a key component to communicate health risks and to reduce tobacco use. More than 39 countries have adopted the recommendations for the use of pictorial warnings [29].

Using the standard protocol to evaluate affective pictures [15], [16], a previous assessment of pictorial health warning labels revealed that depiction of overt body lesions in the pictures evoked the highest emotional responses. Importantly, the presence of smoking cues in a warning significantly weakened its impact on smokers [22]. We based the production of the pictorial warning prototypes on these premises; the physical harms of tobacco were made more overt and vivid, and smoking cues were avoided.

In agreement with the guidelines for implementation of Article 11 [30], a pre-marketing test of pictorial warning prototypes using the standard protocol [15], [16] was performed in Experiment 1. In contrast to the European tobacco warning images, which contained 17% pleasant images (all warnings were subjected to the standard protocol rating system) [31], the Brazilian prototypes were all within the unpleasant range. Smokers judged the prototypes even more aversive than non-smokers. Care to reduce smoking cues in the prototypes may have made them less appealing to smokers. This interpretation is supported by the literature showing that anti-smoking advertisements [32] or even “no-smoking signs” [33] can boost cigarette approach tendencies in smokers.

A recurring concern in the literature (see [11]) is whether the health warnings differently impact subpopulations within a country. Women and low income individuals are of particular concern for tobacco regulators because they represent a current target of tobacco marketing [34]–[36]. Indeed, a higher smoking prevalence among economically underprivileged population groups is now a trend in several countries [36], [37]. For instance, in Brazil, smoking is a problem that reflects the social inequalities of the country; compared to the highest educational levels, the percentage of smokers doubles in populations with low educational levels [27]. In the present study, we found a higher emotional impact of the warning prototypes among women and individuals with lower educational levels. This suggests the potential for the warnings to successfully reach more vulnerable sub-groups, including low-literacy populations (see [38]).

Experiment 2 focused on the warnings stamped on cigarette packs. For smokers, the results revealed a significant cost to bring the pack closer when viewing the warning face compared to the brand face. This result has strong parallels with the literature on implicit motivation in other domains. For instance, pulling a lever towards oneself is facilitated by viewing pleasant stimuli on a screen and is hindered by viewing unpleasant stimuli [23]. In another study, brain recordings showed differential activity when grasping and bringing closer a cylinder filled with pleasant contents (facilitation) compared to unpleasant contents (cost) [24]. Indeed, automatic motivational predispositions elicited by the surrounding environment constitute a basic psychological phenomenon. This process occurs continuously, without intention or awareness, and has strong effects on a person’s decisions and behavior (see [39]). Thorough studies in laboratory settings showed that these automatic predispositions are appropriately linked to the hedonic value of stimuli [23], [25], [40]–[42]. Supported by these experimental psychology premises, we reproduced a more “natural”/daily routine of smokers to explore the interface between health warnings and pack branding, thus reducing the gap between research and practice. There is a vast literature on the public health implications of the gap between research and practice and between a given intervention’s efficacy and its effectiveness in daily practice [11], [43]–[47]. The present study offers a promising approach to narrow the distance between research in controlled settings and real-life circumstances.

Notably, in non-smokers, there was no difference in the latency to bring the pack closer when viewing the warning face compared to the brand face. The lack of an appetitive motivation towards cigarette packs in non-smokers could account for this. Beyond the inherent automatic drive towards packs in smokers, a recent study by Dickter et al. [48] showed that smokers presented a greater perception-action coupling in response to stimuli depicting human interaction with smoking-related cues.

Participants further underwent an evaluative session in Experiment 2 in which they rated the emotional impact and the perceived efficacy against smoking of each pictorial warning. In a telephone survey of smokers, Hammond et al. [8] found that self-reported levels of fear and disgust were associated with the perceived efficacy of Canadian warning labels to induce smoking cessation; however, the authors did not address differences within the set of warnings. In the present work, we compared the levels of aversion for each warning label using a standard protocol [15], [16] that is broadly used in emotion research. There are several advantages to bridging the study of emotional impact of graphic warning labels with experiments on implicit emotional reactions to affective pictures. The literature is extensive, encompassing studies on affective reports and their correlation with physiological reactivity and behavioral modulation, and results have been replicated in different contexts and with different samples [12], [20], [49]–[54].

Some studies compared sets of pictorial warnings with graphic contents (injuries, death) versus sets of pictorial warnings with non-graphic contents, showing that perceptions of efficacy (against smoking) were higher for the graphic sets [11], [55], [56]. Here, we revealed an important link between the rating of an individual warning’s emotional impact and estimations of its efficacy against smoking. Coherent with the cited studies, participants rated the warnings with more explicit graphic content as both more aversive and more effective against smoking (data not shown), demonstrating the value of pictorial warnings with vivid depictions of the harmful effects of tobacco.

### Conclusions

There is unequivocal scientific evidence that tobacco causes death, disease and disability (see [57]). The spread of the tobacco epidemic is a global problem with serious consequences for public health and the Framework Convention on Tobacco Control by the World Health Organization was developed in response to the globalization of this epidemic. Health warnings are one of the key components of this integrated approach to control global tobacco epidemic. The evidence presented in this study adds to the understanding of how implicit responses to pictorial warnings may contribute to behavioral change.

## References

[1] WHO (2008) 2008–2013 action plan for the global strategy for the prevention and control of noncommunicable diseases. Geneva. Available: http://www.who.int/nmh/publications/9789241597418/en/. Accessed 2013 June.

[2] Beaglehole R, Bonita R, Horton R, Adams C, Alleyne G, et al (2011) Priority actions for the non-communicable disease crisis. Lancet 377: 1438–1447 doi: 10.1016/S0140-6736(11)60393-0 21474174 10.1016/S0140-6736(11)60393-0

[3] WHO (2003) Framework convention on tobacco control. Geneva. Available: http://www.who.int/tobacco/framework/en/. Accessed 2012 July.

[4] Frieden TR (2013) Government’s role in protecting health and safety. N Engl J Med 368: 1857–1859 doi: 10.1056/NEJMp1303819 23593978 10.1056/NEJMp1303819

[5] Hiilamo H, Crosbie E, Glantz SA (2012) The evolution of health warning labels on cigarette packs: the role of precedents, and tobacco industry strategies to block diffusion. Tob Control 1–11. doi: 10.1136/tobaccocontrol-2012-050541. 10.1136/tobaccocontrol-2012-050541 PMC3725195 23092884

[6] Wu D (2012) The shock of the new–cigarette pack warnings. Lancet 380: 871 doi:10.1016/S0140-6736(12)61482-2 22959374 10.1016/S0140-6736(12)61482-2

[7] Kees J, Burton S, Andrews JC, Kozup J (2010) Understanding How Graphic Pictorial Warnings Work on Cigarette Packaging. J Public Policy Marketing 29: 265–276 doi:10.1509/jppm.29.2.265

[8] Hammond D, Fong GT, McDonald PW, Brown KS, Cameron R (2004) Graphic Canadian cigarette warning labels and adverse outcomes: Evidence from Canadian smokers. Am J Public Health 94: 1442–1445 doi:10.2105/AJPH.94.8.1442 15284057 10.2105/ajph.94.8.1442 PMC1448469

[9] Peters E, Romer D, Slovic P, Jamieson KH, Wharfield L, et al (2007) The impact and acceptability of Canadian-style cigarette warning labels among U.S. smokers and nonsmokers. Nicotine Tob Res 9: 473–481 doi:10.1080/14622200701239639 17454702 10.1080/14622200701239639

[10] Hammond D (2011) Health warning messages on tobacco products: a review. Tob Control 20: 327–337 doi:10.1136/tc.2010.037630 21606180 10.1136/tc.2010.037630

[11] Hammond D, Wakefield M, Durkin S, Brennan E (2013) Tobacco Packaging and Mass Media Campaigns: Research Needs for Articles 11 and 12 of the WHO Framework Convention on Tobacco Control. Nicotine Tob Res 15: 817–831 doi:10.1093/ntr/nts202 23042986 10.1093/ntr/nts202 PMC3601912

[12] Bradley MM, Codispoti M, Cuthbert BN, Lang PJ (2001) Emotion and motivation I: defensive and appetitive reactions in picture processing. Emotion 1: 276–298 doi:10.1037//1528-3542.1.3.276-298 12934687

[13] Szklo AS, de Almeida LM, Figueiredo VC, Autran M, Malta D, et al (2012) A snapshot of the striking decrease in cigarette smoking prevalence in Brazil between 1989 and 2008. Prev Med 54: 162–167 doi:10.1016/j.ypmed.2011.12.005 22182479 10.1016/j.ypmed.2011.12.005

[14] Ministry of Health (2009) Health Warnings on Tobacco Products - 2009. Rio de Janeiro: INCA.Conprev,Tobacco Control Division.Available: http://www.inca.gov.br/tabagismo/publicacoes/livro_advertencia_ingles.pdf. Accessed 2013 Jun.

[15] Lang PJ, Bradley MM, Cuthbert BN (2008) International affective picture system (IAPS): Affective ratings of pictures and instruction manual. Technical Report A-8 University of Florida, Gainesville, FL.

[16] Bradley MM, Lang PJ (1994) Measuring emotion: the Self-Assessment Manikin and the Semantic Differential. J Behav Ther Exp Psychiatry 25: 49–59 doi:10.1016/0005-7916(94)90063-9 7962581 10.1016/0005-7916(94)90063-9

[17] Azevedo TM, Volchan E, Imbiriba LA, Rodrigues EC, Oliveira JM, et al (2005) A freezing-like posture to pictures of mutilation. Psychophysiology 42: 255–260 doi: 10.1111/j.1469-8986.2005.00287.x 15943678 10.1111/j.1469-8986.2005.00287.x

[18] Pereira MG, Oliveira L, Erthal FS, Joffily M, Mocaiber IF, et al (2010) Emotion affects action: Midcingulate cortex as a pivotal node of interaction between negative emotion and motor signals. Cogn Affect Behav Neurosci 10: 94–106 doi: 10.3758/CABN.10.1.94 20233958 10.3758/CABN.10.1.94 PMC2875262

[19] Pereira MG, Volchan E, de Souza GG, Oliveira L, Campagnoli RR, et al (2006) Sustained and transient modulation of performance induced by emotional picture viewing. Emotion 6: 622–634 doi: 10.1037/1528-3542.6.4.622 17144753 10.1037/1528-3542.6.4.622 PMC2376807

[20] Lang PJ, Bradley MM (2010) Emotion and the motivational brain. Biol Psychol 84: 437–450 doi:10.1016/j.biopsycho.2009.10.007 19879918 10.1016/j.biopsycho.2009.10.007 PMC3612949

[21] Bradley MM, Keil A, Lang PJ (2012) Orienting and emotional perception: facilitation, attenuation, and interference. Front Psychol 3: 493 doi:10.3389/fpsyg.2012.00493 23181039 10.3389/fpsyg.2012.00493 PMC3499912

[22] Nascimento BE, Oliveira L, Vieira AS, Joffily M, Gleiser S, et al (2008) Avoidance of smoking: the impact of warning labels in Brazil. Tob Control 17: 405–409 doi:10.1136/tc.2008.025643 18849316 10.1136/tc.2008.025643

[23] Duckworth KL, Bargh JA, Garcia M, Chaiken S (2002) The automatic evaluation of novel stimuli. Psychol Sci 13: 513–519 doi:10.1111/1467-9280.00490 12430834 10.1111/1467-9280.00490

[24] Oliveira LA, Imbiriba LA, Russo MM, Nogueira-Campos AA, Rodrigues EC, et al (2012) Preparing to grasp emotionally laden stimuli. PLoS One 7: e45235 doi:10.1371/journal.pone.0045235 23024811 10.1371/journal.pone.0045235 PMC3443242

[25] Elliot AJ (2008) Handbook of approach and avoidance motivation. New York: Psychology Press. 584 p.

[26] Oliveira JM, Volchan E, Vargas CD, Gleiser S, David IA (2012) Box for interaction with objects (BIO): A new device to synchronize the presentation of objects with electrophysiological recordings. Behav Res Methods 44: 1115–1120 doi:10.3758/s13428-012-0197-x 22477437 10.3758/s13428-012-0197-x

[27] Instituto Nacional do Câncer, Ministério da Saúde (2010) Brazil global adult tobacco survey report. Available: http://www.who.int/tobacco/surveillance/en_tfi_gats_2010_brazil.pdf. Accessed 2013 Jan.

[28] Fong GT, Hammond D, Jiang YA, Li QA, Quah ACK, et al (2010) Perceptions of tobacco health warnings in China compared with picture and text-only health warnings from other countries: an experimental study. Tob Control 19: i69–i77 doi:10.1136/tc.2010.036483 20935200 10.1136/tc.2010.036483 PMC2976466

[29] Canadian Cancer Society (2010) Cigarette Package Health Warnings: International Status Report. Available: http://www.tobaccolabels.ca/healthwarnings/statusreport. Accessed 2013 Jan.

[30] WHO (2008) Framework Convention on Tobacco Control. Elaboration of guidelines for implementation of article 11 of the convention. Available: http://apps.who.int/gb/fctc/PDF/cop3/FCTC_COP3_7-en.pdf. Accessed 2012 Sep.

[31] Munoz MA, Viedma-Del-Jesus MI, Rossello F, Sanchez-Nacher N, Montoya P, et al (2013) The emotional impact of European tobacco-warning images. Tob Control 22: 123–129 doi:10.1136/tobaccocontrol-2011-050070 22193046 10.1136/tobaccocontrol-2011-050070

[32] Kang Y, Cappella JN, Strasser AA, Lerman C (2009) The effect of smoking cues in antismoking advertisements on smoking urge and psychophysiological reactions. Nicotine Tob Res 11: 254–261 doi:10.1093/ntr/ntn033 19251767 10.1093/ntr/ntn033 PMC2666377

[33] Earp BD, Dill B, Harris JL, Ackerman JM, Bargh JA (2013) No sign of quitting: Incidental exposure to no-smoking signs ironically boosts cigarette-approach tendencies in smokers. J Appl Soc Psychol. In press.

[34] Pirie K, Peto R, Reeves GK, Green J, Beral V (2013) The 21st century hazards of smoking and benefits of stopping: a prospective study of one million women in the UK. Lancet 381: 133–141 doi:10.1016/S0140-6736(12)61720-6 23107252 10.1016/S0140-6736(12)61720-6 PMC3547248

[35] Doxey J, Hammond D (2011) Deadly in pink: the impact of cigarette packaging among young women. Tob Control 20: 353–360 doi:10.1136/tc.2010.038315 21478476 10.1136/tc.2010.038315

[36] Giovino GA, Mirza SA, Samet JM, Gupta PC, Jarvis MJ, et al (2012) Tobacco use in 3 billion individuals from 16 countries: an analysis of nationally representative cross-sectional household surveys. Lancet 380: 668–679 doi:10.1016/S0140-6736(12)61085-X 22901888 10.1016/S0140-6736(12)61085-X

[37] Hiscock R, Bauld L, Amos A, Fidler JA, Munafo M (2012) Socioeconomic status and smoking: a review. Ann N Y Acad Sci 1248: 107–123 doi:10.1111/j.1749-6632.2011.06202.x 22092035 10.1111/j.1749-6632.2011.06202.x

[38] Yong HH, Fong GT, Driezen P, Borland R, Quah AC et al.. (2013) Adult Smokers’ Reactions to Pictorial Health Warning Labels on Cigarette Packs in Thailand and Moderating Effects of Type of Cigarette Smoked: Findings From the International Tobacco Control Southeast Asia Survey. Nicotine Tob Res. doi:10.1093/ntr/nts241. 10.1093/ntr/nts241 PMC3715385 23291637

[39] Bargh JA, Chartrand TL (1999) The unbearable automaticity of being. Am Psychol 54: 462–479 doi:10.1037/0003-066X.54.7.462

[40] Dijksterhuis A, Bargh JA (2001) The perception-behavior expressway: Automatic effects of social perception on social behavior. In: M.Zanna, editors. Advances in Experimental Social Psychology. 1–40.

[41] Bargh JA, Ferguson MJ (2000) Beyond behaviorism: on the automaticity of higher mental processes. Psychol Bull 126: 925–945 doi:10.1037//0033-2909.126.6.925 11107883 10.1037/0033-2909.126.6.925

[42] Sheeran P, Gollwitzer PM, Bargh JA (2013) Nonconscious processes and health. Health Psychol 32: 460–473 doi:10.1037/a0029203 22888816 10.1037/a0029203

[43] Glasgow RE, Lichtenstein E, Marcus AC (2003) Why don’t we see more translation of health promotion research to practice? Rethinking the efficacy-to-effectiveness transition. Am J Public Health 93: 1261–1267 doi:10.2105/AJPH.93.8.1261 12893608 10.2105/ajph.93.8.1261 PMC1447950

[44] Oldenburg BF, Ffrench ML, Sallis JF (2000) Health behavior research: the quality of the evidence base. Am J Health Promot 14: 253–7, iii. doi:10.4278/0890-1171-14.4.253. 10.4278/0890-1171-14.4.253 10915537

[45] Stokols D (1996) Translating social ecological theory into guidelines for community health promotion. Am J Health Promot 10: 282–298 doi:10.4278/0890-1171-10.4.282 10159709 10.4278/0890-1171-10.4.282

[46] Hallfors D, Cho H, Sanchez V, Khatapoush S, Kim HM, et al (2006) Efficacy vs effectiveness trial results of an indicated “model” substance abuse program: implications for public health. Am J Public Health 96: 2254–2259 doi:10.2105/AJPH.2005.067462 16809591 10.2105/AJPH.2005.067462 PMC1698156

[47] Zhu SH, Anderson CM, Tedeschi GJ, Rosbrook B, Johnson CE, et al (2002) Evidence of real-world effectiveness of a telephone quitline for smokers. N Engl J Med 347: 1087–1093 doi:10.1056/NEJMsa020660 12362011 10.1056/NEJMsa020660

[48] Dickter CL, Kieffaber PD, Kittel JA, Forestell CA (2013) Mu suppression as an indicator of activation of the perceptual-motor system by smoking-related cues in smokers. Psychophysiology 50: 664–670 doi:10.1111/psyp.12044 23581629 10.1111/psyp.12044

[49] Lang PJ, Greenwald MK, Bradley MM, Hamm AO (1993) Looking at pictures: affective, facial, visceral, and behavioral reactions. Psychophysiology 30: 261–273 doi:10.1111/j.1469-8986.1993.tb03352.x 8497555 10.1111/j.1469-8986.1993.tb03352.x

[50] Bradley MM, Lang PJ (2007) The International Affective Picture System (IAPS) in the study of emotion and attention. In: Coan JA, Allen JJB, editors. Handbook of Emotion Elicitation and Assessment. Oxford University Press. 29–46.

[51] Lang PJ (1995) The emotion probe. Studies of motivation and attention. Am Psychol 50: 372–385 doi:10.1037/0003-066x.50.5.372 7762889 10.1037//0003-066x.50.5.372

[52] Sabatinelli D, Lang PJ, Keil A, Bradley MM (2007) Emotional perception: correlation of functional MRI and event-related potentials. Cereb Cortex 17: 1085–1091 doi:10.1093/cercor/bhl017 16769742 10.1093/cercor/bhl017

[53] Schupp HT, Cuthbert BN, Bradley MM, Cacioppo JT, Ito T, et al (2000) Affective picture processing: the late positive potential is modulated by motivational relevance. Psychophysiology 37: 257–261 doi:10.1111/1469-8986.3720257 10731776

[54] Olofsson JK, Nordin S, Sequeira H, Polich J (2008) Affective picture processing: an integrative review of ERP findings. Biol Psychol 77: 247–265 doi:10.1016/j.biopsycho.2007.11.006 18164800 10.1016/j.biopsycho.2007.11.006 PMC2443061

[55] Thrasher JF, Carpenter MJ, Andrews JO, Gray KM, Alberg AJ, et al (2012) Cigarette warning label policy alternatives and smoking-related health disparities. Am J Prev Med 43: 590–600 doi:10.1016/j.amepre.2012.08.025 23159254 10.1016/j.amepre.2012.08.025 PMC3504356

[56] Hammond D, Thrasher J, Reid JL, Driezen P, Boudreau C, et al (2012) Perceived effectiveness of pictorial health warnings among Mexican youth and adults: a population-level intervention with potential to reduce tobacco-related inequities. Canc Causes Contr 23 Suppl 157–67 doi:10.1007/s10552-012-9902-4 10.1007/s10552-012-9902-4 PMC4586036 22362058

[57] Mathers CD, Loncar D (2006) Projections of global mortality and burden of disease from 2002 to 2030. Plos Med 3: e442 doi:10.1371/journal.pmed.0030442 17132052 10.1371/journal.pmed.0030442 PMC1664601

